# Using a Deep Learning Model to Explore the Impact of Clinical Data on COVID-19 Diagnosis Using Chest X-ray

**DOI:** 10.3390/s22020669

**Published:** 2022-01-16

**Authors:** Irfan Ullah Khan, Nida Aslam, Talha Anwar, Hind S. Alsaif, Sara Mhd. Bachar Chrouf, Norah A. Alzahrani, Fatimah Ahmed Alamoudi, Mariam Moataz Aly Kamaleldin, Khaled Bassam Awary

**Affiliations:** 1 Department of Computer Science, College of Computer Science and Information Technology, Imam Abdulrahman Bin Faisal University, Dammam 31441, Saudi Arabia; iurab@iau.edu.sa (I.U.K.); 2170007790@iau.edu.sa (S.M.B.C.); 2170005400@iau.edu.sa (N.A.A.); 2170007764@iau.edu.sa (F.A.A.); 2170007806@iau.edu.sa (M.M.A.K.); 2 School of Computing, National University of Computer and Emerging Sciences, Islamabad 44000, Pakistan; chtalhaanwar@gmail.com; 3 Radiology Department, King Fahd Hospital of the University, Imam Abdulrahman Bin Faisal University, Dammam 31441, Saudi Arabia; Hssaif@iau.edu.sa (H.S.A.); kbawary@iau.edu.sa (K.B.A.); 4 National Center for Artificial Intelligence (NCAI), Saudi Data and Artificial Intelligence Authority (SDAIA), Riyadh 12391, Saudi Arabia

**Keywords:** COVID-19, pneumonia, chest X-ray (CXR), deep learning (DL), clinical data

## Abstract

The coronavirus pandemic (COVID-19) is disrupting the entire world; its rapid global spread threatens to affect millions of people. Accurate and timely diagnosis of COVID-19 is essential to control the spread and alleviate risk. Due to the promising results achieved by integrating machine learning (ML), particularly deep learning (DL), in automating the multiple disease diagnosis process. In the current study, a model based on deep learning was proposed for the automated diagnosis of COVID-19 using chest X-ray images (CXR) and clinical data of the patient. The aim of this study is to investigate the effects of integrating clinical patient data with the CXR for automated COVID-19 diagnosis. The proposed model used data collected from King Fahad University Hospital, Dammam, KSA, which consists of 270 patient records. The experiments were carried out first with clinical data, second with the CXR, and finally with clinical data and CXR. The fusion technique was used to combine the clinical features and features extracted from images. The study found that integrating clinical data with the CXR improves diagnostic accuracy. Using the clinical data and the CXR, the model achieved an accuracy of 0.970, a recall of 0.986, a precision of 0.978, and an F-score of 0.982. Further validation was performed by comparing the performance of the proposed system with the diagnosis of an expert. Additionally, the results have shown that the proposed system can be used as a tool that can help the doctors in COVID-19 diagnosis.

## 1. Introduction

The novel coronavirus (COVID-19), also known as SARS-CoV-2, is a severe acute respiratory syndrome. The virus first emerged in China in December 2019. Up to 7 October 2021, about 236,533,988 people were affected by this virus [1]. This pandemic outbreak has spread around the world. In addition, a new strain of coronavirus was reported in the southeast of England in September 2020. Additionally, in December 2020 it became more common. The new trunk spreads faster and more continuously when compared to the previous version [2]. According to the BBC, the new strain was first detected in September, and in November around a quarter of the cases reported in London were the new variant [3]. In addition, several cases have been reported in other countries. This outbreak is having a devastating impact on the global economy and the lives of individuals.

The outbreak of the pandemic requires early diagnosis and treatment of the virus. The gold standard method for diagnosing COVID-19 is the Reverse Transcriptase Polymerase Chain Reaction (RT-PCR) test. The test requires specialized medical equipment and a healthcare professional. Due to the manual method of collecting the nasal or throat swab, there is an increased risk of the infection spreading. It is also time consuming and has low sensitivity [4,5]. Due to the unavailability of a large number of these specialized tool kits and medical personnel, there is a need for other non-invasive automated diagnostic methods with higher accuracy.

One of the alternative methods of diagnosing COVID-19 is to use chest X-rays (CXR) and chest CT scans. The devices required for this imaging are widely used around the world. The advantages of X-rays over CT scan include quick access and control of interaction in the hospital. CXR can be used to detect early signs of viral infection, even before the appearance of the other symptoms such as cough, fever, shortness of breath, etc.

An experienced radiologist is required for accurate interpretation and diagnosis. However, the exponential increase in the number of cases worldwide makes an automated detection and diagnosis method necessary. In addition, in a report dated 11 June 2020, the WHO recommends the use of CXR if RT-PCR toolkits are not available [6]. Likewise, the high costs associated with the toolkit motivates the use of CXR for COVID-19 diagnosis. Several attempts have been made to combat COVID-19, including the development of an automated and reliable diagnostic method that can support the diagnostic methods used in the clinical setting. In addition, artificial intelligence (AI), in particular machine learning (ML) and deep learning (DL) techniques, have proven to be an effective tool for the automated detection and diagnosis of diseases using medical imaging [7,8,9]. These automated diagnostic models will assist healthcare professional in making a diagnosis.

The proposed study is structured in such a way that Section 2 presents the relevant studies and Section 3 deals with the material and the methods used in the study. Section 4 presents the experimental setup and the results. Section 5 contains the conclusion.

## 2. Related Studies

Several attempts have been made to apply ML and DL using clinical features, medical imaging, blood tests, cough, etc. [7,10,11,12]. However, CXR is one of the most widely used tools for diagnosing COVID-19.

A study [13] was conducted to examine the effects of lung segmentation prior to classifying COVID-19 using DL. They used CXR and Lungs ultrasound (LUS), and found that combining transfer learning (TL) with segmentation improved the model’s performance. Four TL methods such as ResNet34, ResNet50, ResNet152, and VGG16 were used. In addition, the ensemble method was performed by combining the results of the previous four TL methods. The study achieved an accuracy of 0.905 using an ensemble technique.

Similarly, another study [14] was carried out to find the correlation between the CXR, LUS, and clinical data of the COVID-19 patients under treatment. They found a strong correlation between CXR, LUS, and clinical data of hospitalized patients to predict severity. In addition, the authors found in [15] the correlation between clinical features, CXR and the laboratory results for the patients in the critical stage. They found the correlation between all investigative measures for all COVID-19 confirmed patients.

Similarly, in [16] the authors compared the performance of the DL-based model using CXR with the diagnosis of the experienced radiologist using real data from Italian hospitals to predict the severity of the patient. The prognostic value predicted by the DL model is comparable to the score of the Radiographic Assessment of Lung Edema (RALE). All studies discussed above point to the importance and correlation of the CXR with the other diagnostic procedures and motivate the use of the CXR for the diagnosis and prognosis of COVID-19 patients [16].

Recently, the Deep COVID-XR model was proposed by [17], an ensemble of CNN, including DenseNet-121 (17), ResNet-50 (18), InceptionV3 (19), InceptionV3-ResnetV2 (20), Xception (21), Efficient Net-B2 (22) to compare the performance of the model with the experienced radiologist. The result of the proposed model was compared with the diagnosis made by five chest radiologists. They achieved an accuracy of 0.83 and an AUC of 0.90.

In addition, the authors [18] proposed a model known as DeTraC using the TL models. The model consists of three stages, first the features were extracted from the CXR, individual features were entered into the TL models and later this information was combined again to make the prediction. AlexNet was used to extract the features and principal component analysis (PCA) for feature reduction; that has successfully overcome the irregularities in the data. VGG-19 gave the highest result with an accuracy of 0.931.

Furthermore, another study [19] compared the performance of three TL models Inception V3, XceptionNet and ResNet using Kaggle’s open source CXR. The study achieved the highest accuracy of 0.9797 with XceptionNet. In addition, another study was done with several open source CXRs [20] to compare the performance of a number of TL models such as DenseNet121, ResNet50, VGG16, and VGG19 for diagnosing COVID-19. They found the similar performance of the VGG16 and VGG 19 models. The model achieved the highest accuracy of 0.993 with VGG 16 and 19.

The comparison was made using several conventional ML models such as Support Vector Machine (SVM), Logistic Regression (LR), Decision Tree (DT), Naive Bayes (NB), and K Nearest Neighbor (KNN) and TL models such as VGG-16, VGG-19, InceptionV3, MobileNet-V2, ResNet 50, and DenseNet 121 for diagnosing COVID-19 with CXR [21]. The study achieved the highest accuracy of 0.985. They found that the initial preprocessing did not affect diagnostic performance. The aforementioned study [13], however, found that preprocessing technique was important for diagnostic performance.

The study [22] proposed CV19-Net as a DL based model and found that the proposed model can improve radiological interpretation performance in COVID-19 diagnosis and achieve an AUC of 0.92. They used an ensemble of 20 DL models. The study achieved the significant results, but the number of models in the ensemble is huge and the proposed model was computationally intensive.

Similarly, another author aimed to develop a predictive model using ML and DL techniques to estimate mortality risk based on some demographic information and the patient’s X-ray [23]. The researchers conducted two experiments. First, they used CNN with TL techniques to predict mortality risk using CXR. This model has performed well in mitigating the effects of the small amount of data available. While in the second experiment the researchers worked on was to combine the CNN together with other patient demographics, including age and gender, as input to a final machine learning model that acts as the second and final layer. This layer improved the goodness of fit and the efficiency of the first layer’s predictive power. The researchers used two sources of data to create their data set. The first is the COVID-chestxray-dataset, a GitHub with X-ray and basic clinical data from 209 patients. The second source is the Spanish Society of Medical Radiology, SERAM, COVID-19 data. The researchers manually extracted 12 segments. The combined data set thus comprises 221 patients. Each patient has CXR, gender, age, hospital location, X-ray view, and X-ray offset. This data set was then divided into 65% training, 17.5% validation and 17.5% testing. They also expanded the data set to create additional data. The results of the first experiment without data expansion are: an AUC of 0.93 for training, an AUC of 0.87 for validation, and an AUC of 0.85 for testing. With data extension, the results were an AUC of 0.93 for training, an AUC of 0.93 for validation, and an AUC of 0.94 for testing. When a second layer was added, the results were an AUC of 0.99 for training, an AUC of 1 for validation, and an AUC of 1 for testing.

Moreover, Minaee et al. [24] analyzed 5000 CXR images from COVID Chest X-ray and ChexPert data sets. The pretrained DL models such as ResNet18, DenseNet121, SqueezeNet, and ResNet50 were trained. All models were refined with 100 epochs along with 20 batches, Adam optimizer, and a learning rate of 0.001. A down sampling technique was applied to all images and reshaped to 224,224. Most of the models achieved a sensitivity value of 0.98 (±0.3) and a specificity value of around 0.90. These results demonstrate the tremendous importance of X-rays in diagnosing COVID-19 patients. The researchers suggest using larger data sets to obtain more reliable results and estimates.

Lopez et al. [25] developed a COVID-XNet model using several CXR dataset for COVID-19 and healthy patients. The images were collected from BIMCV-COVID-19+ [26], Cohen et al. [27] and PadChest dataset [28]. Feature extraction and classification was performed using CNN model. The study achieved an accuracy of 0.94. In addition another study [29] used the pre-trained DL models to classify the CXR as COVID-19 positive or negative using nine open source dataset. Initially some preprocessing techniques was performed to clean the CXR by identifying the relevant area, i.e., lungs segmentation. The study achieved an accuracy of 0.97. The current study developed a more generalized model and achieved better performance compared to the later study. Due to the use of the larger dataset as compared with Lopez at al. Similarly, Nikolaou et al. [30] used EfficientNetB0 pretrained model using 15,153 CXR. The dataset suffers from class imbalance. Data augmentation was performed to handle imbalance. The study achieved an accuracy of 0.95. Furthermore, Baltazar et al. [31] performed a comparative analysis of several pretrained models. The aim of the study was to analyze the hyperparameter tunning impact on the pretrained model performance. InceptionV3 outperformed the other models to distinguish among the normal, COVID-19 and non-COVID-19 pneumonia CXR with an accuracy of 0.96.

Keidar et al. [32] implemented deep neural networks (DNN) aimed at classifying COVID-19 chest X-rays, in addition to a tool that can retrieve similar CXR images according to the provided CXR image. The study used 1384 CXRs from COVID-19 patients and 1024 CXRs from non-COVID-19 patients. The images were augmented, normalized, and segmented before being used in the model. The classifier was an ensemble of different deep learning networks such as ReNet50, VGG16, ReNet34, CheXpert, and ResNet152. The model achieved an accuracy of 0.903, a sensitivity of 0.905, a specificity of 0.90 and an AUC of 0.96. The main limitation of the study was that the model did not consider the existence of medical illnesses and their impact on the outcome of the model.

Nishio et al. [33] investigated the impact of data augmentation technique on the classification of CXRs as COVID-19 pneumonia, non-COVID-19 pneumonia and healthy. They used two open-source datasets, i.e., github CXR repository and RSNA dataset. Five TL models were used such as VGG16, ResNet50, MobileNet, DenseNet121, and EfficientNet. Experiments were conducted with and without data augmentation. The study found that VGG16 with the augmented data produce the highest accuracy of 0.837.

Additionally, in [26] the researchers focused on classifying the images from COVID-19 CXRs using two sets of data, COVID-X-ray-5k, and the other data set was the COVIDetectioNet dataset. They suggested a methodology using two phases: First a deep CNN network is used to extract the features from the images, then it is fed into the Extreme Learning Machines (ELM) network to provide real-time learning, using the Chimp Optimization Algorithm (ChOA) with it, to ensure the reliability of the ELM model. The proposed solution outperformed the benchmark and achieved an accuracy of 0.99 and 0.98 using the COVIDetectioNet and COVID-X-ray-5k datasets, respectively.

Nevertheless, most studies are done with CXR only and do not consider clinical features or vital signs. To the best of our knowledge, no study is available that integrates the clinical data and the CXR to predict COVID-19. In the proposed work, we try to fill this gap by examining the effects of demographics, vital signs, and comorbidity in diagnosing COVID-19 with CXR. We proposed a method to integrate the features extracted from clinical data and CXR images with deep learning (DL). We are trying to examine the impact of clinical data on predicting COVID-19 using CXR. In addition, the performance of the proposed system was further validated by comparing the diagnosis of the model with the expert (doctor) diagnosis. There are few studies in the literature that have conducted the comparison among the doctor and the system diagnosis.

## 3. Materials and Methods

In this section we first describe the description of the data set used in the study. Next, we present the methodology used and finally discuss the evaluation matrix.

### 3.1. Data Acquisition and Description

The data were collected from King Fahad University Hospital, Dammam, KSA (IRB No. 2020-09-237). The project was approved by the Deanship of Scientific Research (DSR) at Imam Abdulrahman bin Faisal University. The data set contains 270 patients (48 healthy and 222 COVID-19 positive). The healthy category indicates the patients that have symptoms but not pneumonia. The data set includes clinical, demographic data, and CXR images of the patients. Table 1 contains the description of the attributes in the data record.

The dataset contains 16 attributes of categorical or numerical datatype. Some of the features were not significant and have been removed. To perform the exploratory data analysis, mean(µ) and standard deviation(σ) were used for numeric attributes, while frequency and *p*-value were used for categorical attributes. In addition, several visualizations were used to determine the distribution of the data. Table 2 contains the exploratory data analysis of the numerical attributes.

**Figure 1:**
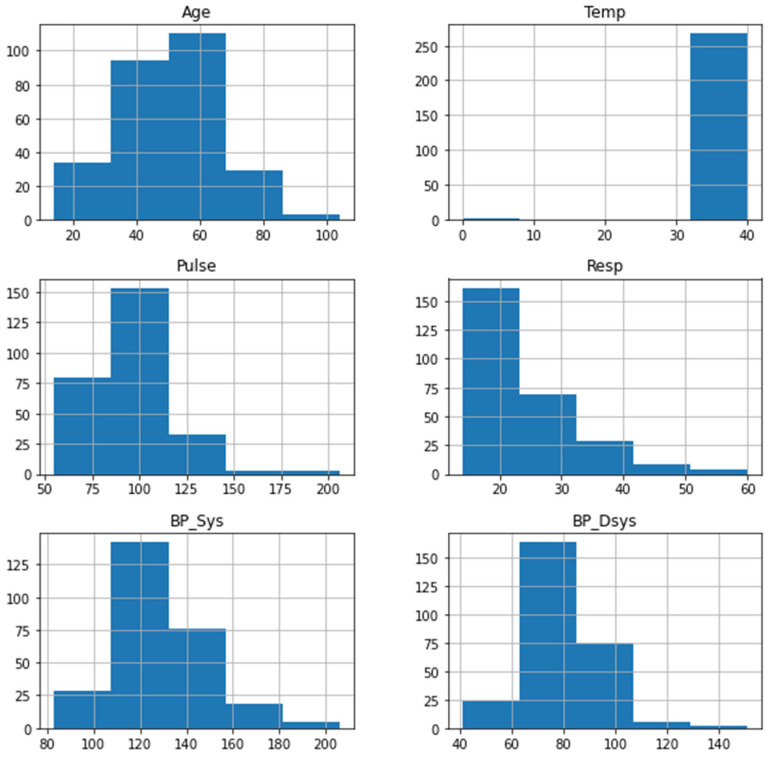
Exploratory analysis of the numeric attributes.

**Figure 2:**
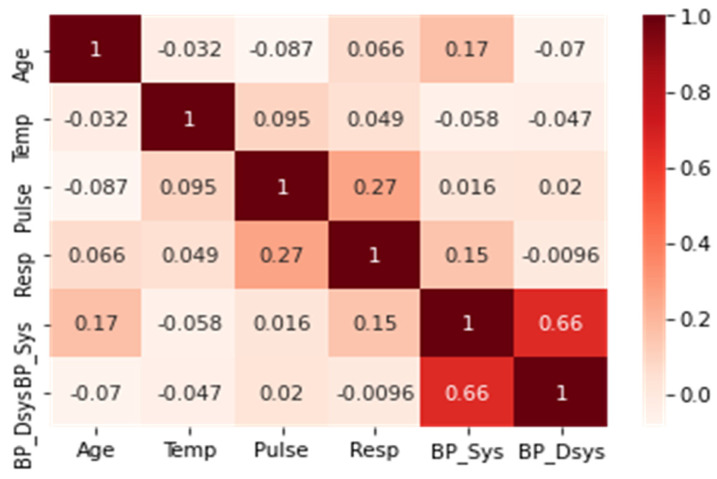
Correlation of the numeric attributes in the dataset.

Figure 1 shows the distribution of the numerical attributes. The age feature shows an almost normal distribution, most of the temperature values are in the range of 30 to 40, the pulse and the respiratory rate both showed a right-skewed distribution, and the diastolic and systolic blood pressure both show an almost normal distribution. Figure 2 shows the correlation of numerical attributes. The heat map shows the correlation between six numerical variables. The higher the correlation value between two features means that the two features express the same information and one of them can be used. In Figure 2, a correlation value of 0.66 was recorded between both systolic and diastolic blood pressure.

Furthermore, Table 3 represents exploratory analysis of categorical attributes. The table contains the description of the categorical features of the dataset in terms of frequency and *p*-value. *p*-value was calculated using chi-square, with the α = 0.05. Results showed that the gender and SOB has strong association with COVID-19 positive. Similarly, according to the dataset, most of the COVID-19 positive patients has cough. The dataset contains huge number of male patients when compared with the female. Furthermore, 148 patients’ samples in the dataset have a cough, and among those patients 129 patients were COVID-19 positive. Similarly, 139 patients have SOB symptoms and 122 patients with SOB were COVID-19 positive. However, COVID-19 positive patients’ samples in the dataset have only 42% of the patients with Diabetes Mellitus, 37.8% patients with hypertension, 19.8% with cardiac disease, 18.3% dyslipidemia, and 14% chronic kidney disease, respectively.

### 3.2. Image Augmentation

In order to train the DL model with all aspects of the data and to have the model learn effectively, image augmentation techniques were applied to the training set. The images are synthetically augmented by applying various image transformation techniques such as flipping (both horizontal and vertical), rotation with 30, shifting (width and height with 0.2), cropping with central_fraction = 0.3, blurred images with brightness_range = [0.5,1.5], zoom range set to 0.2, rescale set 1/255, fill mode set to ‘nearest’, and shearing set to 0.2. The images were set to a shape of 300 or 224 pixels in the case 2 and case 3 models described below.

### 3.3. Deep Learning Model

Deep Neural Networks (DNNs) are a form of neural networks whose basic architecture includes an input layer, a series of hidden layers, and an output layer. DNNs are usually feed-forward networks (FFNN) in which data is propagated from the input layer to the output layer. The layers are described below:

*Input Layer:* This layer is where all input values (*x_n_*) enter the neural network. Moreover, each input has a weight (*w_n_*) applied to it. Moreover, a bias (*b*) is added to the entire summation. This summation ($\sum_{i=1}^{n}\left(x_{i}\times w_{i}\right)+b$) is then sent to the hidden layer, where an activation function is applied.

*Hidden Layer:* This layer applies *f*, an activation function, to the summation it receives from the input layer. The result is then sent to all nodes in the subsequent hidden layer. A deep neural network may contain multiple hidden layers. Each hidden layer takes the output of the previous layer as input. The output of each layer can be represented as Equation (1):

(1) $$y_{n}=f\;\left(\overset{n}{\underset{i=1}{\sum }}\left(x_{i}\times w_{i}\right)+b\right)\;$$

*Output Layer:* In this layer, the output value is determined from the hidden layers. The goal is to reduce the output error rate. Therefore, if an output is determined, then back propagation is used to reduce the error rate of the output. Backpropagation is carried out using a method known as Gradient Descent. This method attempts to adjust the weights that are applied to the inputs in the input and hidden layers. The new weights (*W_n_*) are found using Equation (2):

(2) $$W_{n}=w_{n}-\alpha \left(\frac{\delta \;error}{w_{n}}\right)$$

For the classification of CXR images, we used EfficientNetB7 transfer learning with ImageNet weights, which is one of the renowned pre-trained CNN architectures built by Google. As DL models requires huge amounts of data to avoid overfitting, which makes them computationally intensive. Thus, the transfer learning is commonly technique where we trained the model for one task and reusing it for another related task. EfficientNet-B7 model is a latest architecture and trained on the ImageNet dataset.

The aim of the study is to explore the impact of the clinical data on the prediction of COVID-19 using CXR images, therefore, three set of experiments were performed. In the proposed study, three DL models were developed for three different cases. In the first case, clinical data was used for the prediction of COVID-19, while in the second case prediction was performed using CXR images. Finally, in the third case, clinical data and CXR images were combined to make the prediction. The details of the DL models for each case are discussed below.

#### 3.3.1. Case 1: Deep Learning Model for Clinical Data

**Figure 3:**
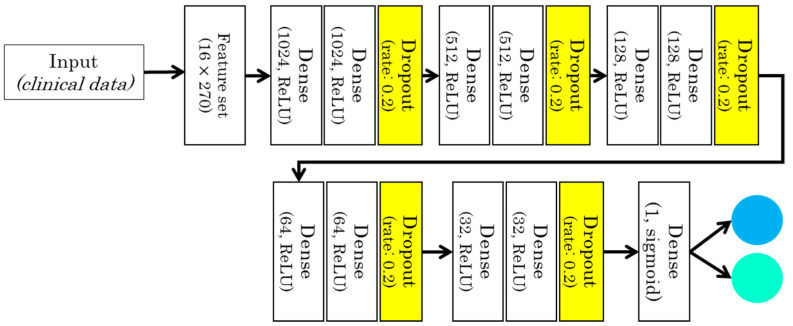
Deep Learning model for the clinical data (case 1).

The proposed DL model has thirteen fully connected dense layers that take sixteen variables as input. The block structure is adopted, where each block contains two hidden layers. So, in total we have six blocks, the number of neurons in each block is 1024, 512, 256, 128, 64, 32 and one neuron in the output layer as shown in Figure 3. Each block is followed by a dropout layer with a dropout rate of 20%. This layer allows the model to learn more robustly to avoid overfitting. Furthermore, the rectified linear unit (ReLU) activation function was used for all dense layers except the last dense layer which uses the sigmoid activation function. Sigmoid activation function was used because the problem we are targeting is a binary class, whether a patient is COVID-positive or healthy. The ReLU activation function has Equation (3):

(3) R(x)=max(0,x)

If the input to the activation function is negative, the output is zero, and thus, the node will not be activated. This makes the neural network sparser and more efficient. On the other hand, the sigmoid activation function has Equation (4):

(4) $$\delta \left(x\right)=\frac{1}{1+e^{-x}}\;$$

The sigmoid function provides us with a value that is between [0, 1]. This is useful because we can use the resulting value as a probability for a particular class. If the value is closer to one, we can classify the input instance as part of the positive class. Otherwise, we classify the input instance as part of the negative (healthy) class. For model optimization, we used the Adam optimization algorithm with a learning rate of 0.001. Moreover, the loss was calculated using binary cross-entropy and the accuracy metric was used to evaluate the model’s accuracy. In model training, we used 200 epochs with a batch size of 64.

#### 3.3.2. Case 2: Deep Learning Model for CXR

Convolutional Neural Network (CNN) is a type of DL model designed to learn spatial hierarchies of features from images. Since features can appear anywhere in the image, the CNN is designed to extract local features from the images, making image processing more efficient. CNN consists mainly three layers, (i) convolution, (ii) pooling, and (iii) fully connected layers. The convolution layer calculates the similarity among the small patches from the image and the kernel, while the pooling layer groups the pixel values that are close and combines them into one pixel and the fully connected layer classifies the extracted features. Numerous CNN architectures have been developed for image classification using the TL techniques. DenseNet, ResNet, VGG19, and EfficientNet are a few examples of them that are pre-trained TL models with ImageNet datasets.

**Figure 4:**
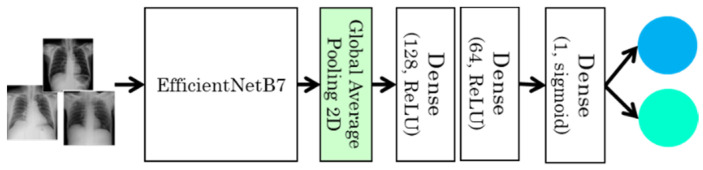
Deep Learning model for the chest X-ray (case 2).

For case 2 DL model, we used the pretrained EfficientNetB7 using the ImageNet’s weights. For the fully connected classification layers, a Global Average Pooling layer is used followed by three dense layers as shown in Figure 4. The first two dense layers includes 128 and 64 neurons and the ReLU activation function, while the output layer contain one neuron and the sigmoid activation function. Again, for this model, we used the Adam optimizer with a learning rate of 0.001, binary cross-entropy as a loss function, accuracy as the evaluation metric, and epoch with thirty.

#### 3.3.3. Case 3: Deep Learning Model for Fusion of Clinical Data and CXR

**Figure 5:**
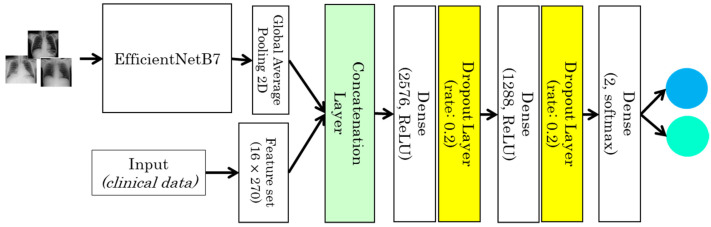
Joint-fusion model for chest X-ray and clinical data (case 3).

For this model, we used a fusion technique in which multiple inputs modalities are merged into a single feature vector before it is feed into a single FFNN. In this study we have used join-fusion, in which the features are learned from the images via the EfficientNetB7 transfer learning model and fused with the clinical data in the fusion layer before being fed them into an FFNN as shown in Figure 5. Simple concatenation is used to fuse the learned imaging and clinical features. Furtherly, the FFNN has three dense layers separated by a dropout layer with a dropout rate of 20%. The first two dense layers includes 2576 and 1288 neurons and ReLU as an activation function, while the output dense layer contain two neurons and softmax as an activation function. The final model uses the Adamx optimizer with a learning rate of 0.001, a beta1 of 0.9, a beta2 of 0.999, and an epsilon of 1e-07. We also used categorical cross-entropy as a loss function and the evaluation metric of accuracy.

### 3.4. Evaluation Metrics

The performance of the proposed models was assessed using several standard scoring measures such as accuracy, precision, recall, and F-score. The study targets a binary-class classification problem, and using 2 × 2 size for confusion matrix to output the four values: True Positive (TP), False Positive (FP), False Positive (FP), and False Negative (FN), which are used to calculate the following measures [34]:

*Accuracy:* It measures the ratio of correctly predicted patients to the total samples as shown in Equation (5).

(5) $$Accuracy=\frac{TP+TN}{TP+TN+FP+FN}$$

*Recall:* It measures the ratio of the correctly predicted COVID-19 positive patients over the actual positive patients as shown in Equation (6).

(6) $$Recall\;=\frac{TP}{TP+FN}\;$$

*Precision:* It measures the ratio of correctly predicted COVID-19 positive patients over the predicted positive patients as shown in Equation (7).

(7) $$Precision\;=\frac{TP}{TP+FP}\;$$

*F-score:* It is measured by finding the harmonic mean of both the precision and recall values as shown in Equation (8).

(8) $$F-Sccore=\frac{2\times Prescision\times Recall}{Prescision+Recall}$$

## 4. Experiments and Results

The models have been trained on NVIDIA GeForce RTX-2060 SUPPER with 8GB of memory while the CPU specification is Intel i7-9700F and 16GB of RAM, which are used for the tasks related to augmentation and classifications. The study is implemented using the Python 3.8.5 language and the TensorFlow 2.5 library for the DL models. Moreover, we used k-fold cross-validation with k set to 5.

**Figure 6:**
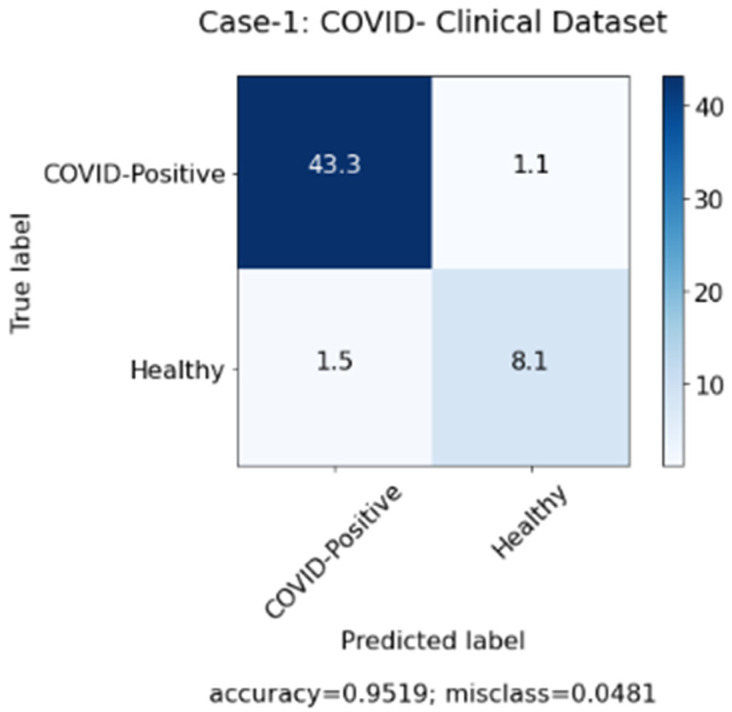
Confusion matrix for case 1 (clinical data).

**Figure 7:**
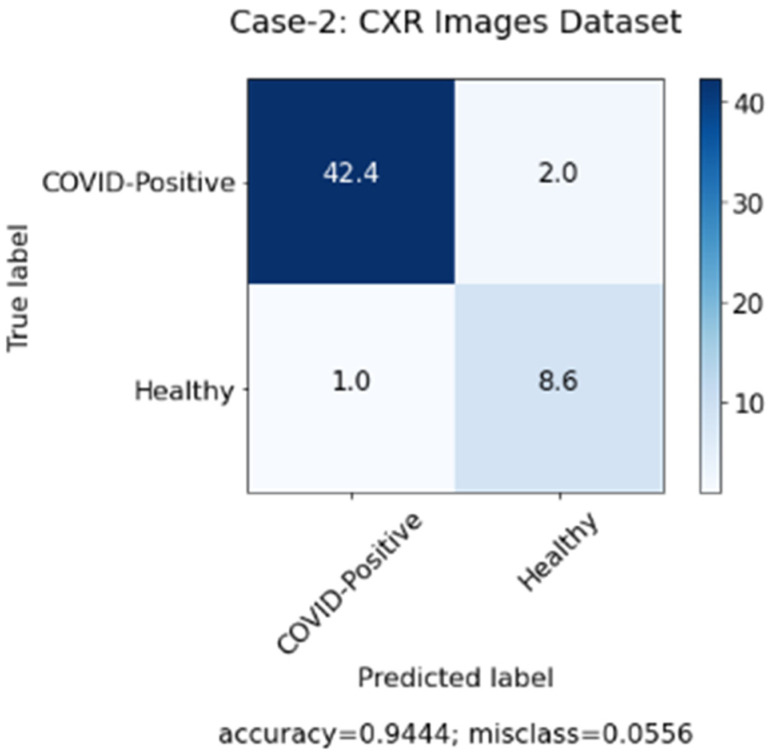
Confusion matrix for case 2 (chest X-ray (CXR)).

**Figure 8:**
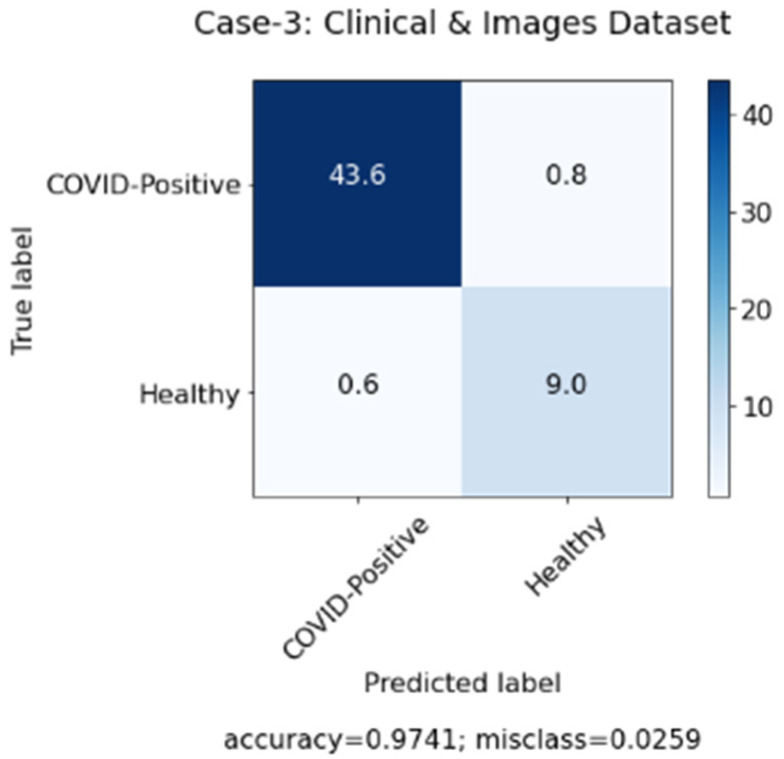
Confusion matrix for case 3 (clinical data and chest X-ray (CXR)).

In this study, we aimed to investigate the effect of clinical data on automated COVID-19 diagnosis using CXR. As mentioned in Section 3, three DL models have been created that aim to diagnose COVID-19 patients in three experiments. The first experiment was conducted with clinical data from the patients only and the second experiment was performed with CXR from patients, while, in the last experiment, we applied a hybrid approach and combined both sets of data (clinical data and CXR). Table 4 summarizes the results achieved of the proposed models based on three different cases. Similarly, Figure 6, Figure 7 and Figure 8 represent the confusion matrix for all the three cases.

Table 4 above shows that the hybrid approach showed significant improvements in terms of all the performance measures, especially in terms of accuracy with a value of 0.970. Moreover, the high values of precision and recall scores indicate good performance of the model in terms of false positive and false negative rates. The 0.986 value of the recall shows that the model aims to minimize the false-negative rates so that it does not show a high loss of COVID-19 infected cases. Whereas the precision value of 0.978 shows that the model also minimizes the false positive rates, so it does not show a high misclassification of COVID-19 infected cases. Furthermore, since DL models require an immense number of images to achieve acceptable performance, the second case where only images were used showed the worst results due to the shortage of COVID-19 patients’ CXR in the used dataset. Therefore, we believe that combining CXR images with clinical data can be considered a solution for health authorities to diagnose COVID-19 patients in an efficient and quick manner.

Furthermore, some of the examples of the COVID-19 positive and the healthy patients from the dataset are included in Table 5. The clinical data represent the attributes sequence, i.e., gender, age, body temperature, pulse rate, respiratory rate, blood pressure systolic, blood pressure diastolic, shortness of breath, cough, other symptoms, diabetes mellitus, hypertension, cardiac disease, dyslipoproteinemia, chronic kidney disease, and other chronic diseases.

**Figure 9:**
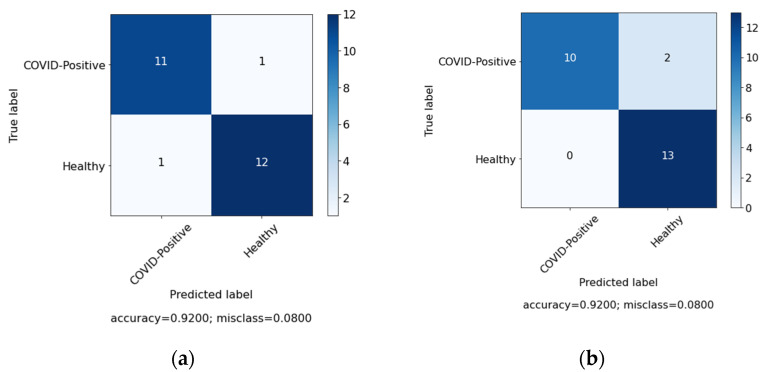
Diagnosis comparison for case 1 (clinical data). (**a**) Expert diagnosis. (**b**) System diagnosis.

**Figure 10:**
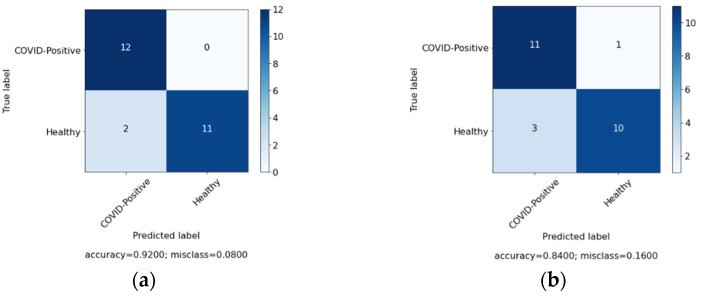
Diagnosis comparison for case 2 (CXR). (**a**) Expert diagnosis. (**b**) System diagnosis.

**Figure 11:**
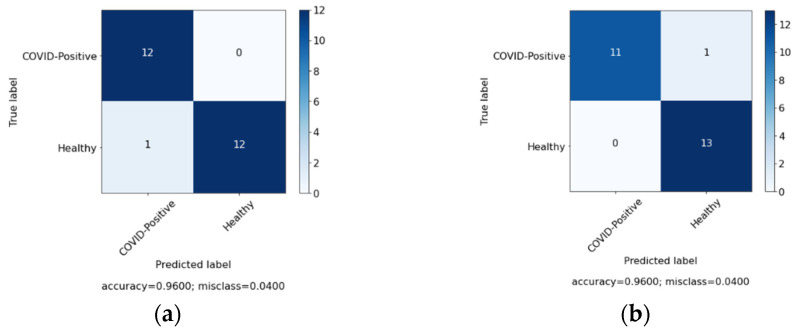
Diagnosis comparison for case 3 (fusion). (**a**) Expert diagnosis. (**b**) System diagnosis.

In order to further validate the performance of the proposed system, comparison was made among the system and the doctor diagnosis for some of the patients. Figure 9, Figure 10 and Figure 11 represents the confusion matrix. Figure 9a represent the confusion matrix for the diagnosis conducted by the expert (doctor) and Figure 9b represents the confusion matrix for the diagnosis conducted by the proposed system. Similarly, Table 6 contains the evaluation measure for the diagnosis comparison. For comparison 25 new records were selected, among them 12 were COVID-19 positive and 13 were healthy patients.

As seen from Table 6, integration of the clinical data with the CXR has produced better diagnosis performance when compared with the case 1 and case 2. Furthermore, there is a difference in the diagnosis performance in case 2, because of the limitation of shortage of the CXR used to train the DL model. The proposed system cannot replace the doctors but can assist them in the diagnosis of COVID-19.

Despite of the significant results achieved from the proposed model. The study also suffers from some limitation. Due to the availability of one expert and unavailability of the large number of patients records, the validation was performed on 25 patients’ samples.

## 5. Conclusions

Overall, the study proposed three main models. The first model was developed using clinical data, the second one was developed using CXR, and the last one was developed using both clinical data and CXR. The study used a real dataset that was obtained from King Fahad University Hospital, KSA, that contains 270 records. The study addressed the gap in developing a model that would enable the prediction of COVID-19 using both clinical data and CXR images. Overall, the model achieved the highest performance on the combination of clinical data and CXR with an accuracy of 0.970, a recall of 0.986, a precision of 0.978, and an F-score of 0.982. The results of the proposed model were further validated by comparing the performance of the system with the expert diagnosis. The system has shown similar performance except for case 2 due to the shortage of the CXR used to train the DL models. However, the results have proved the significance of the clinical data with the CXR in the diagnosis of COVID-19. Nevertheless, the study suffers from the data imbalance. The imbalance is due to the fact that there were very few patients in the hospital that were suspected as COVID-19 with the CXR. However, those patients were later diagnosed as COVID-19 negative. For the future recommendation, the proposed models need to be further investigated using multi-center data and validated on large number of records. Similarly, the study needs to be further extended by including lungs ultrasound (LUS).

## Figures and Tables

**Table 1:** Attribute description of the dataset.

Feature Type	Feature Name	Description	Data Type
Demographic	Gender	Patient’s Gender	Categorical
	Age	Patient’s age at the time of diagnosis	Numerical
Symptoms	Temp	Patient’s body temperature at the time of diagnosis	Numerical
	Pulse	Patient’s pulse rate at the time of diagnosis	Numerical
	Resp	Patient’s respiratory rate at the time of diagnosis	Numerical
	BP_Sys	Patient’s systolic blood pressure at the time of diagnosis	Numerical
	BP_Dsys	Patient’s diastolic blood pressure at the time of diagnosis	Numerical
	SOB	Does the patient have Shortness of Breath?	Categorical
	Cough	Does the patient have Cough?	Categorical
	Others	Does the patient have any other symptoms?	Categorical
comorbidities	DM	Does the patient have Diabetes Mellitus?	Categorical
	HTN	Does the patient have Hypertension?	Categorical
	Cardiac	Does the patient have any Cardiac problem?	Categorical
	DLP	Does the patient have Dyslipidemia?	Categorical
	CKD	Does the patient have chronic kidney diseases?	Categorical
	Others	Does the patient have any other chronic disease?	Categorical

**Table 2:** Statistical description of the numeric attributes.

Feature Name	Mean (µ) ± Standard Deviation (σ)
Age	50 ± 16
Temp	37.1 ± 4
Pulse	96.7 ± 20.99
Resp	25.13 ± 7.87
BP_Sys	129 ± 19.7
BP_Dsys	79 ± 14.11

**Table 3:** Statistical analysis of the categorical attributes.

Feature Name	Frequency		*p*-Value
Gender	Male (187)	Female (83)	<0.001
SOB	Yes (139)	No (131)	0.04
Cough	Yes (148)	No (122)	0.053
DM	Yes (211)	No (59)	0.997
HTN	Yes (198)	No (72)	0.408
Cardiac	Yes (146)	No (124)	0.077
DLP	Yes (142)	No (128)	0.094
CKD	Yes (134)	No (136)	0.071

**Table 4:** Performance comparison of the proposed DL models for three different cases.

Experiment Scenario	Datatype	Accuracy	Recall	Precision	F-Score
Case 1	Clinical data	0.952	0.964	0.977	0.971
Case 2	CXR	0.944	0.981	0.951	0.966
Case 3	Clinical data + CXR	0.970	0.986	0.978	0.982

**Table 5:** Sample CXR and clinical data for the COVID-19 positive and healthy cases.

Category	CXR	Clinical Data
COVID-19 Positive	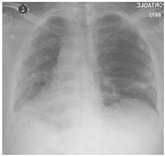	Male, 68, 37, 70, 27, 129, 76, Y, Y, asymptomatic, Y, Y, Y, Y, N, anemia
	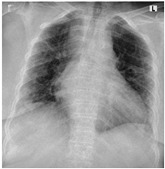	Female, 74, 38.2, 78, 20, 134, 53, Y, Y, diarrhea, Y, Y, N, Y, N, bph
Healthy	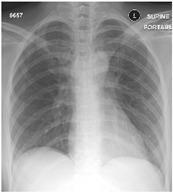	Male, 57, 36.6, 63, 20, 123, 87, N, N, dizz, Y, Y, Y, N, N, anemia
	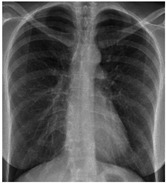	Female, 36, 37, 102, 20, 103, 66, Y, N, headache, N, N, N, N, N, sickle cell

**Table 6:** Diagnosis comparison among the doctor and the system for the three different cases.

Case	Diagnosis Mode	Accuracy	Recall	Precision	F1
Case 1	Expert	0.920	0.917	0.917	0.917
	System	0.920	0.833	1.000	0.909
Case 2	Expert	0.920	1.000	0.857	0.923
	System	0.840	0.917	0.786	0.846
Case 3	Expert	0.960	1.000	0.923	0.960
	System	0.960	0.917	1.000	0.957

## Data Availability

The data are not available due to ethical reasons.
